# DiRT v2.0: An Optimized Pipeline for Detecting Dicistronic tRNA-mRNA Transcripts in Plants

**DOI:** 10.21769/BioProtoc.5754

**Published:** 2026-06-20

**Authors:** Fei Zheng, Lakshay Anand, Roberta Magnani, Carlos Rodríguez M. López, Rakesh David

**Affiliations:** 1 School of Agriculture, Food and Wine, Adelaide University, Adelaide, SA, Australia; 2 Environmental Epigenetics and Genetics Group, Department of Horticulture, Martin-Gatton College of Agriculture, Food and Environment, University of Kentucky, Lexington, KY, USA

**Keywords:** Transcriptomic analysis, Novel transcriptomic features, Motif analysis, Bioinformatics, Dicistronic transcripts, tRNA-mRNA

## Abstract

The canonical role of transfer RNAs (tRNAs) in protein synthesis has been extensively characterized; however, recent studies have uncovered novel functions for tRNA as a mediator of long-distance signaling in plants. Several studies have identified dicistronic tRNA-mRNA transcripts that contain a tRNA gene and an adjacent protein-coding gene (PCG) that are transcribed as a single unit. These transcripts are associated with RNA systemic mobility through the plant’s vascular tissues, potentially acting as non-cell-autonomous signaling messengers in coordinating development and stress responses. Here, we report a computational pipeline to detect dicistronic tRNA-mRNA transcripts from short-read next-generation RNA-sequencing datasets; to our knowledge, this is the only established pipeline for the systematic identification of such candidates in plants. The dicistronic RNA transcript version 2 (v2) described here improves on the earlier version DiRT v1 by expanding the repertoire of dicistronic transcripts detected to include tRNA-like structures (TLS) as well as functional tRNAs, which were already supported in the pipeline. The updated protocol also includes detection of dicistronic tRNA or TLS sequences within genomic features such as untranslated regions (UTRs). The accurate detection of both tRNAs and UTR-embedded tRNA-like sequences (TLS) is critical, as these RNA structures have been reported to function as mediators of long-distance RNA mobility. Furthermore, as NGS datasets are prone to sequencing artifacts and potential DNA contamination, we improved the pipeline’s statistical robustness by including read coverage of flanking intronic regions as a baseline control. To account for potential DNA contamination during RNA-seq library preparation, detected tRNA-mRNA transcripts are deemed as putatively dicistronic only if the coverage of their intergenic region is significantly higher (Student’s t-test, FDR < 0.05) than flanking intronic regions. Furthermore, the updated pipeline allows this statistical test to be applied to intronless and single-intron genes. Using this updated protocol, we identified novel tRNA and TLS dicistronic transcripts in both grapevine (*Vitis* spp. Ruggeri 140) and *Arabidopsis thaliana* datasets and validated *in vitro* using RT-PCR. We provide a fast and reliable method to detect dicistronic transcripts that can be applied to any short-read RNA-sequencing dataset, fast-tracking the functional characterization of these newly emerging transcripts.

Key features

• DiRT version 2.0 (v2) provides a reliable and improved bioinformatics workflow to identify dicistronic tRNA-mRNA transcriptomic features in plants.

• The updated workflow improves detection of tRNA-like structures (TLS) and UTRembedded dicistronic tRNA–mRNA candidates.

• Applies Student’s *t*-test (FDR < 0.05) and base pair coverage to validate transcript continuity, utilizing neighboring introns as the appropriate biological background control.

• DiRT v2 supports prediction of dicistronic candidates from intronless and singleintron protein coding genes (PCG).

## Graphical overview


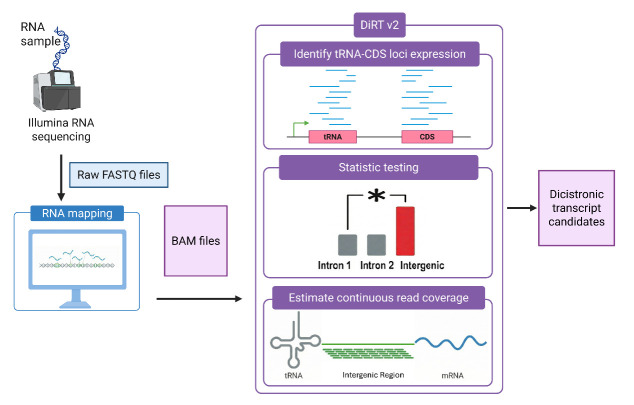


**Bioinformatic workflow.** RNA samples are sequenced by the Illumina NovaSeq X Plus. Following standard mapping of reads to a reference genome, BAM files generated are processed by DiRT v2, which identifies dicistronic tRNA-mRNA transcript candidates based on three criteria: (1) co-expression of both the tRNA and the adjacent protein coding gene (CDS); (2) statistically significant expression in the intergenic region compared to background noise (introns); and (3) continuous read coverage spanning the junction between the tRNA and mRNA (intergenic region).

## Background

Dicistronic poly(A)-mRNA-tRNA transcripts occur when a protein-coding gene (PCG) and its adjacent tRNA gene are transcribed as a single transcriptional unit. Prior work shows that such co-transcribed mRNA-tRNA molecules can move systemically through the plant to target distant tissues [1]. The transgenic removal of tRNA or tRNA-like structure (TLS) sequences from these dicistronic transcripts renders them immobile, suggesting that movement is controlled by signals within the tRNA structure, sequence, or both [1]. Furthermore, as part of the co-transcribed units, mRNAs are translated to functional proteins in the target tissue, suggesting tRNAs or TLSs can facilitate the movement of these transcripts, potentially expanding their role in non-cell autonomous signaling pathways [1,2]. In field-grown commercial grapevines, the expression patterns of dicistronic tRNA-mRNAs were linked to the geographical location of the vineyards as well as the tissue type analyzed [3], which indicates that the expression patterns of these transcripts may be related to the microclimate, such as temperature or light, in different vineyards. We previously reported the development of a reusable computational workflow to reliably detect dicistronic tRNA-mRNA transcripts from RNA-seq datasets. The dicistronic tRNA-mRNA transcript (DiRT) workflow identified dicistronic tRNA-mRNA transcripts in multiple vascular species but not in non-vascular lineages, suggesting that their co-transcription may be related to phloem transportation of mRNA into distant tissues [3].

Although the majority of nuclear-encoded genes in plants are expressed as monocistronic units, an increasing number of studies report that plants utilize polycistronic and dicistronic transcripts to coordinate complex regulatory networks. For example, plant small nucleolar RNAs (snoRNAs) are frequently co-transcribed with adjacent tRNAs as dicistronic precursors [4]. In *Arabidopsis thaliana* and rice, these snoRNAs are transcribed from an upstream tRNA promoter to form dicistronic tRNA-snoRNA transcripts, which are subsequently cleaved into independent, functional tRNA and snoRNA [4]. Similarly, microRNAs (miRNAs) have been shown to cluster together in the rice genome and transcribed as a single polycistronic transcript [5]. These findings underscore the genome-wide prevalence of eukaryotic polycistronic transcription. While the biological roles of these co-transcribed units are still emerging, accurately capturing these complex transcripts directly from widely available short-read transcriptomic data remains a bottleneck. The first version of the DiRT pipeline v1 was developed to identify dicistronic tRNAs located upstream or downstream of mRNAs [3], but did not include tRNA-related sequences present in the untranslated regions (UTRs) of protein-coding genes. Indeed, many of the TLSs that share some structural similarities with canonical tRNAs but are non-functional as a translational adaptor are found localized to UTR regions of protein-coding genes [1]. Transgenic studies using TLSs derived from a tRNA in which specific functional domains were deleted were still able to confer long-distance mobility of their co-transcribed mRNA [1,2]. In *Arabidopsis*, TLS motifs located in the UTR of *CK1* play a key role in regulating gene function by acting as a mobility motif for the *CK1* transcript [1]. Furthermore, studies of turnip yellow mosaic virus (TYMV) and brome mosaic virus have demonstrated that TLS in the UTR regions of viral genes can mimic endogenous plant tRNAs to trigger systemic mobility through the phloem [6–8]. Together, these findings highlight the biological relevance of TLS and the importance of computational workflows to reliably detect dicistronic UTR-localized TLS sequences, as they may be functionally important in mobile signaling pathways in plants. Here, we address this gap by introducing DiRT pipeline v2, an enhanced version that incorporates the detection of tRNA-related sequences within mRNA UTRs.

Dicistronic tRNA-mRNA transcripts are also known to exhibit low expression levels compared to monocistronic nuclear-encoded protein-coding genes based on RNA-seq data [1,3], indicating the need for internal expression controls to distinguish biologically relevant transcript signals from technical noise or sequencing artifacts. The DiRT v1 pipeline relied on active transcription in the intergenic region between the tRNA and mRNA as indicative of co-transcription and compared the read coverage in this region with the two closest introns in the dicistronic protein-coding genes. tRNA-mRNA combinations were selected for further analysis in the workflow if the read coverage in the intergenic region was significantly higher than the intron-level coverage. However, this strategy excluded analyzing potential dicistronic tRNA-mRNA transcripts from protein-coding genes with one or no introns. As a significant proportion of protein-coding genes in plant species have one or no introns [9,10], excluding these genes may cause significant bias in the analysis of potential dicistronic tRNA-mRNA transcripts.

To overcome this bias, we modified the workflow for genes with no introns. Here, the average sequencing depth of the intergenic region [between the tRNA/TLS and the coding sequence (CDS) forming the given transcript] is compared with the top depth obtained for the first two introns from the closest protein-coding gene. For the analysis of dicistronic protein-coding genes with a single intron, an intron from the next closest flanking gene was used for sequencing depth comparison. After implementing these adjustments to control for minimum expression threshold levels, RNA coverage of each base pair of the intergenic region was estimated. Candidates that show both intergenic region continuous coverage and sequencing depth above background noise were classified as dicistronic tRNA-mRNA transcripts.

## Materials and reagents

**Biological material**

1. *Vitis* Ruggeri 140

2. *Arabidopsis thaliana* Col-0 wild-type

**Reagents**

1. Spectrum^TM^ Plant Total RNA kit (Sigma-Aldrich, UNSPSC Code: 41105501)

2. NEBNext^®^ Poly(A) mRNA magnetic isolation module (New England Biolabs, catalog number: E7490S)

3. NEBNext^®^ Ultra^TM^ II Directional RNA library prep with sample purification beads (New England Biolabs, catalog number: E7775S)

4. SuperScript^TM^ III first-strand synthesis system (Invitrogen, catalog number: 18080051)

5. DreamTaq PCR master mixes (2×) (Thermo Scientific, catalog number: K1081)

## Software and datasets

DiRT v2 pipeline implementation is dependent on software packages in Linux, R, and RStudio environment to identify dicistronic tRNA-mRNA transcripts (Tables 1 and 2). Two RNA-seq datasets were used as examples for implementation of the DiRT v2 pipeline (Table 3).

**Table 1.** Linux environment

Type	Software/dataset/resource	Version	Access (free/paid)
Software 1	AdapterRemoval [11]	v2.3.3	free
Software 2	FastQC [12]	v0.12.1	free
Software 3	HISAT2 [13]	v2.2.1	free
Software 4	Samtools [14]	v1.22.1	free
Software 5	tRNAscan-SE [15]	v2.0	free
Software 6	BEDtools [16]	v2.30.0	free

**Table 2.** R and R Studio environment

Type	Software/dataset/resource	Version	Access (free/paid)
Environment	R	v4.5.2	free
Environment	RStudio	2025.09.2 Build 418	free
Package 1	Bioconductor [17]	v3.22	free
Package 2	tidyverse [18]	v2.00.0	free
Package 3	data.table	v1.18.2.1	free
Package 4	GenomicAlignments [19]	v1.46.0	free
Package 5	rtracklayer [20]	v1.70.1	free
Package 6	GenomicFeatures [21]	v1.62.0	free
Package 7	Txdbmaker [22]	v1.62.0	free
Package 8	GeneOverlap [23]	v1.46.0	free

**Table 3.** RNA-seq datasets and reference genomes

Species (tissue type)	RNA-seq dataset	Reference genome source file	GFF annotation version and file	Download resources	Recommended local file path
*Vitis* Ruggeri 140 (leaf)	Data 1: https://doi.org/10.5281/zenodo.20421456	PNT2T_ref.fasta	Version 5.1: PN40024_5.1_on_T2T_ref_with_names.gff3	https://grapedia.org/files-download/	~/DiRT_v2/data/grapevine/
*Arabidopsis thaliana* (whole seedling)	Data 2: PRJEB32714 (SRA)	Arabidopsis_thaliana. TAIR10.dna.toplevel.fa	Ensembl Plants v59: Arabidopsis_thaliana.TAIR10.59.gff3	https://plants.ensembl.org/Arabidopsis_thaliana/Info/Index	~/DiRT_v2/data/arabidopsis/

## Procedure

**A. Bioinformatic pipeline for RNA alignment**

**Step 0: Software installation**

Install Linux software:

# Create the environment with your validated tool versions

conda create -n dirt_v2 -c bioconda -c conda-forge adapterremoval=2.3.3 fastqc=0.12.1 hisat2=2.2.1 samtools=1.22.1 trnascan-se=2.0 bedtools=2.30.0 -y

# Activate the environment

conda activate dirt_v2

Install R and RStudio following the installation instructions at RStudio Desktop - Posit.

Then install R packages:

if (! require("BiocManager", quietly = TRUE))

 install.packages("BiocManager")

pkgs <- c("tidyverse",

 "data.table",

 "GenomicAlignments",

 "rtracklayer",

 "GenomicFeatures",

 "txdbmaker",

 "biomaRt",

 "GeneOverlap")

BiocManager::install(pkgs)

**Step 1: Pre-processing and quality control**

Process raw fastq files from Illumina RNA sequencing data using AdapterRemoval v2 to remove sequencing adapters and short-length reads (minimum length < 100 bps). Post-trimming, verify quality using FastQC.

AdapterRemoval --file1 "RNASeq_R1.fq.gz" --file2 "RNASeq_R2.fq.gz" --minlength 100 --trimns –trimqualities –gzip --output1 "RNA.R1.trimmed.fq.gz" --output2 "RNA.R2.trimmed.fq.gz" --singleton "RNA.singleton.trimmed.fq.gz"

fastqc -o $data/fastqc $data/*

# RNA-Seq_R1.fq.gz RNA-Seq_R2.fq.gz represent read 1 and read 2 from pair end RNA-seq samples.

Following adaptor removal and FastQC analysis, a quality control step is required to detect and filter out any potential genomic DNA contamination that may have inadvertently been co-extracted during RNA preparation and carried over to the sequencing library. To ensure robust downstream analysis of dicistronic candidates, process RNA-seq data using the R/Bioconductor package CleanUpRNAseq tool that provides a range of methods for detecting sample-level genomic DNA contamination based on the user’s choice of RNA-seq library protocol. CleanUpRNAseq includes a range of correction methods for stranded or unstranded RNA-seq data if the detected genomic contamination levels are considered to be high [24].

The CleanUpRNAseq tool and the RNA-seq correction methods can be accessed in the GitHub link: https://github.com/haibol2016/CleanUpRNAseq/tree/devel.

**Step 2: Alignment and filtering**

Align trimmed reads to the reference genome using HISAT2 with default parameters. To ensure high-quality downstream analysis, filter alignments using samtools (-F 4) to retain only successfully mapped reads. Resulting BAM files are then sorted and indexed for R-based analysis.

hisat2 -p 8 \

 -x "Genome_index" \

 -1 "RNA.R1.trimmed.fq.gz" \

 -2 "RNA.R2.trimmed.fq.gz" \

 --rna-strandness RF \

 --dta \

 --mp 4,2 \

 --score-min L,0,-0.4 \

 --summary-file "RNA_summary.txt" \

 | samtools view -@ 8 -b -F 4 - \

 | samtools sort -@ 8 -m 1G -o "RNA.sorted.bam" -

samtools index -@ 8 "RNA.sorted.bam"

**B. DiRT v2.0 pipeline to identify dicistronic tRNA-mRNA transcripts**

To identify dicistronic transcripts, a genome assembly and genome annotation of the target species are required. First, scan the genome assembly for tRNA genes and tRNA-like sequences (TLS) using tRNAscan-SE v2 [15]. Retain all highly confident predictions (internal score > 20), including pseudo-tRNA genes and tRNA genes lacking an identifiable anticodon (tRNA-undetNNN). These sequences are retained as tRNA and TLS candidates because truncated tRNAs have been reported to participate in mRNA mobility and are considered functionally relevant [1,2].

Next, import the genome annotation file (GFF) into the R environment and extract the coding sequence (CDS) coordinates for each annotated gene. Define the CDS region for each gene by selecting the minimum and maximum genomic coordinates across all annotated CDS entries belonging to that gene. Extract CDS coordinates directly from the GFF file within R.

Identify candidate tRNA-mRNA genomic pairs using Bedtools closest. For each mRNA CDS, determine the nearest neighboring tRNA or TLS sequence identified in the previous step. Retain these neighboring pairs for downstream analysis.

Quantify RNA read coverage for each candidate tRNA-mRNA pair using the GenomicRanges and GenomicAlignments packages in R. To be classified as expressed, it is recommended that a candidate meet a minimum threshold of 10 read counts in at least three biological replicates. For each expressed tRNA–mRNA combination deemed as expressed, calculate the average read coverage across the intergenic region and introns.

As read coverage in the intergenic region between the transcribed tRNA and mRNA would indicate co-transcription, the next step assesses the significance of read coverage in this region to further mitigate the effects of genomic contamination or stochastic background noise that are not reproducible across biological replicates. Using data from a minimum of three biological replicates, perform a Student’s t-test to determine whether read coverage in the intergenic region is significantly higher than the coverage observed in the introns of the flanking protein-coding genes. For genes containing two or more introns, compare the intergenic region with the introns of the dicistronic protein-coding gene. For genes containing a single intron or no introns, use the introns of the nearest neighboring gene as the background control for read coverage.

**Figure 1.**
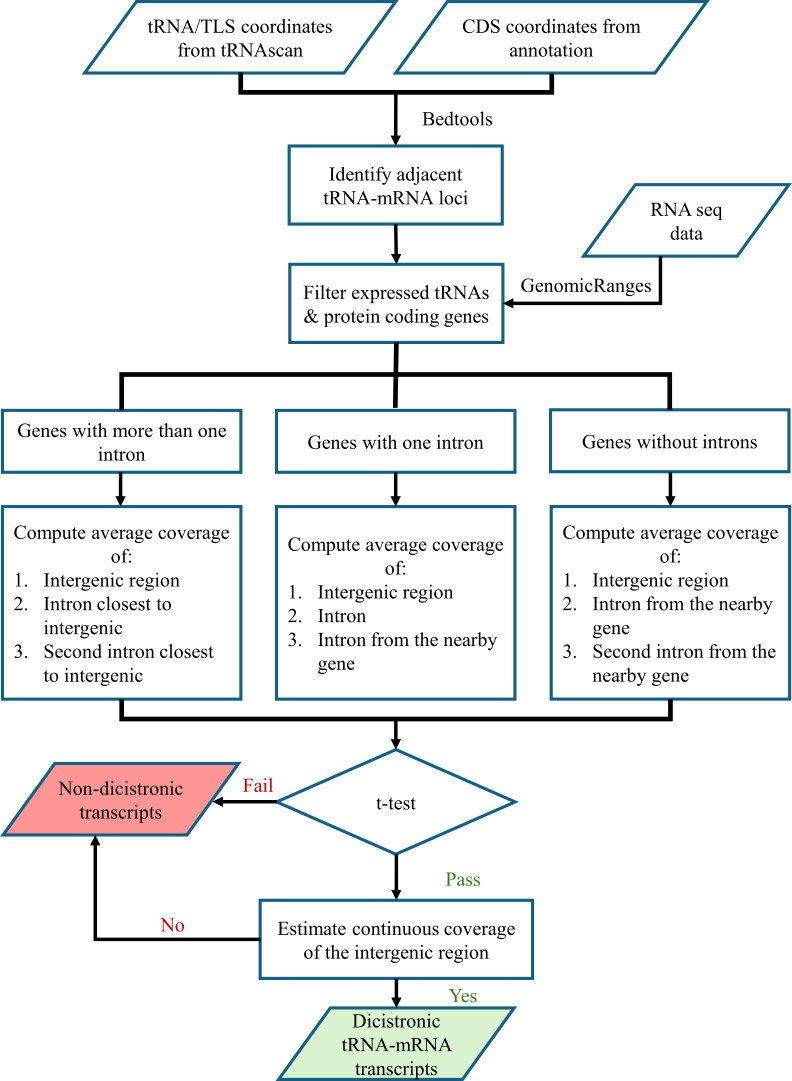
DiRT v2 workflow. The tRNA gene coordinates are annotated by tRNAscan-SE, and the protein-coding gene (PCG) coding sequence (CDS) coordinates are derived from genome annotations. Expression pairs of tRNA genes and their adjacent PCGs are filtered and categorized according to their intron numbers: genes with multiple introns, one intron, or without introns. For each category, average RNA read coverage of the intergenic region is compared with representative introns from the same or nearby genes (Student’s t-test). Candidates with intergenic read coverage significantly higher than intron read coverage (p ≤ 0.05) and that have a continuous intergenic coverage are classified as dicistronic tRNA-mRNA transcripts.

After performing the statistical test, adjust the resulting p-values using the Benjamini–Hochberg (BH) procedure to control the false discovery rate (FDR). Retain candidates that pass the statistical threshold (FDR < 0.05) for further analysis. Next, evaluate single-base read coverage across the intergenic region between the tRNA and mRNA CDS. Classify candidates that show continuous read coverage at every base position across this region as dicistronic tRNA-mRNA transcripts (Figure 1).

To account for variability across biological replicates, the DiRT v2 pipeline evaluates continuous coverage using two complementary criteria. First, candidates exhibiting complete intergenic coverage independently in each replicate are recorded as TRUE in the continu.cov column. Second, because dicistronic tRNA-mRNA transcripts are often expressed at low levels [1,3], reads from all replicates are pooled to increase detection sensitivity. Candidates displaying continuous intergenic coverage in the merged dataset are recorded as TRUE in the combined.cov column.

**B1. Code for each key step in DiRT v2 pipeline**

**1. Extract CDS and tRNA genomic coordinates.** Retrieve the genomic coordinates for all annotated tRNAs and the coding sequences (CDS) of protein-coding genes (PCGs) from the standard reference genome annotation file (see General notes and Troubleshooting). tRNA genes’ coordinates that are annotated by tRNA-scan SE v2.0 are used for finding potential dicistronic CDS_tRNA coordinates.

{R}:

# Load gene annotation

gene.info.from.gff <- read_delim("Reference_genome.gff3",

 delim = "\t", col_names = c("gene.chr","V2","gene.biotype","gene.start","gene.end","V6","gene.strand","V8","gene.id")

) %>%

 select(gene.chr, gene.start, gene.end, gene.id, gene.strand, gene.biotype) %>%

 mutate(across(c(gene.start, gene.end), as.numeric)) %>%

 arrange(gene.chr, gene.start) %>%

 na.omit()

# Extract the longest CDS start end position per gene

CDS.start.end.for.gene <- gene.info.from.gff %>%

 filter(gene.biotype == "CDS") %>%

 mutate(gene.id = sub(".*;Parent=(.*)_t\\d+.*", "\\1", gene.id)) %>%

 group_by(gene.id) %>%

 summarise(

 gene.chr = first(gene.chr),

 gene.start = min(gene.start),

 gene.end = max(gene.end),

 gene.strand = first(gene.strand),

 gene.biotype = first(gene.biotype),

 .groups = "drop"

 ) %>%

 select(gene.chr, gene.start, gene.end, gene.id, gene.strand, gene.biotype) %>%

 arrange(gene.chr, gene.start)

# Get tRNA information from tRNAscan_SE

{Linux}

tRNAscan-SE -E -o tRNA.txt Reference_genome.fasta

awk 'NR > 3 {print $1 "\t" $3 "\t" $4 "\t" $5 "\t" $9 "\t" $6}' tRNA.txt > tRNA.bed

{R}

# Load tRNA information

tRNA.info <- read_delim("tRNA.bed", delim = "\t", col_names = FALSE)

colnames(tRNA.info) <- c("chr", "start", "end", "amino_acid", "score", "anticodon")

# Add a tRNA id

tRNA.info <- tRNA.info %>%

 dplyr::mutate(

 number = dplyr::row_number(),

 tRNA_id = paste0("tRNA", number, "-", amino_acid, anticodon))

tRNA.fixed <- tRNA.info %>%

 mutate(

 # Create valid BED Coordinates (Always Min first, Max second)

 bed_start = pmin(start, end),

 bed_end = pmax(start, end),

 # Create the Strand Column based on the original direction: If original Start > End, it is Negative (-)

 bed_strand = ifelse(start < end, "+", "-"),

 # Placeholder score (BED requires a score in col 5 usually, 0 is fine)

 bed_score = 0) %>%

 # Order: Chr, Start, End, Name, Score, Strand

 dplyr::select(chr, bed_start, bed_end, tRNA_id, bed_score, bed_strand)

Identify the potential dicistronic CDS_tRNA loci. Using the BEDTools closest function, identify the nearest upstream and downstream PCG from each tRNA coordinate. The distance between the tRNA and PCGs is not restricted (see General notes and Troubleshooting).

{Linux}

samtools faidx Reference_genome.fasta

cut -f1,2 Reference_genome.fasta.fai > Reference_genome.txt

## Find closeest gene to a tRNA for both behind and after by BEDTools

bedtools closest -id -a tRNA_sorted.txt -b CDS.start.end.for.gene.sorted.txt -g Reference_genome_sorted.txt -D ref > Closest_tRNA_gene_upS.bed

bedtools closest -iu -a tRNA_sorted.txt -b CDS.start.end.for.gene.sorted.txt -g Reference_genome_sorted.txt -D ref > Closest_tRNA_gene_downS.bed

**2. Estimate tRNA and mRNA sequencing depths.** Use the GenomicRange R package to quantify tRNA gene and PCG sequencing depth from the input bam files. Unnormalized read counts are retained for downstream statistical tests.

GRange_tRNA <-

makeGRangesFromDataFrame(tRNA.info,

 keep.extra.columns = TRUE,

 ignore.strand = FALSE,

 seqinfo = Seqinfo,

 seqnames.field = "tRNA.chr",

 start.field = "tRNA.start",

 end.field = "tRNA.end",

 strand.field = "tRNA.strand",

starts.in.df.are.0based = FALSE)

## Count reads for each tRNA GRange from your RNA-seq bam files

# specify Samples

bam.dir <- "bam_folder_path/" # Dir for bamfiles

bamfiles <- list.files(bam.dir, pattern=".bam$", full.names = TRUE)

SampleName <- basename(bamfiles)

counts <- lapply (bamfiles,function(bamfile){

baifile <- paste0(bamfile,".bai")

bam.input <-

readGAlignments(bamfile,

 index = baifile,

 use.names = TRUE,

 param = ScanBamParam(which = GRange_tRNA))

countOverlaps(GRange_tRNA, bam.input,

 maxgap=0L, minoverlap=1L,

 type=c("any"),

 select=c("all"),

 ignore.strand=TRUE)

})

names(counts) <- SampleName

counts <- do.call(cbind,counts)

tRNA.counts <- cbind(tRNA.info,counts)

## filter commonly expressed tRNA

# filter expressed tRNA

tRNA.expressed.AllSample <- rowSums(dplyr::select(tRNA.counts,6:8) >= 1) == 3 # the number 6:8 (means column6 to column8) are sample columns, we want to find tRNA gene expressed at least 1 RNA reads in all replicates.

# return logical value for all read count column

sum(tRNA.expressed.AllSample)

common.tRNA <- tRNA.counts[tRNA.expressed.AllSample,]

tRNA.expressed.CDS.combo <- filter(tRNA.CDS.combo, tRNA.id %in% common.tRNA$tRNA.id)

# prepare gene data

gene.counts <- tRNA.expressed.CDS.combo %>%

 dplyr::select(chr, gene.start, gene.end, gene.id, gene.strand) %>%

 unique()

# build up GRange for the gene

GRange_gene <-

makeGRangesFromDataFrame(gene.counts,

 keep.extra.columns = TRUE,

 ignore.strand = TRUE,

 seqinfo = Seqinfo,

 seqnames.field = "chr",

 start.field = "gene.start",

 end.field = "gene.end",

 strand.field = "gene.strand",

 starts.in.df.are.0based = FALSE)

## Count reads for each gene GRange from ```bam``` files.

counts <- lapply (bamfiles,function(bamfile){baifile <- paste0(bamfile,".bai")

bam.input <-

readGAlignments(bamfile,

 index = baifile,

 use.names = TRUE,

 param = ScanBamParam(which = GRange_gene))

countOverlaps(GRange_gene, bam.input,

 maxgap=0L, minoverlap=1L,

 type=c("any"),

 select=c("all"),

 ignore.strand=TRUE)

})

names(counts) <- SampleName

counts <- do.call(cbind,counts)

gene.counts <- cbind(gene.counts,counts)

# filter expressed gene

gene.expressed.AllSample <- rowSums(dplyr::select(gene.counts, 6:8) >= 1) == 3 # same requirement as above

sum(gene.expressed.AllSample)

common.gene <- gene.counts[gene.expressed.AllSample,]

tRNA.CDS.expressed.combo <- filter(tRNA.expressed.CDS.combo, gene.id %in% common.gene$gene.id)

**3. Identify dicistronic candidates with one or no introns in the protein-coding gene.** Determine introns' coordinates by using PCG’s exon information from the annotation file.

# Find genes with no intron (one exon)

expressed.gene.exon_2 <- tgene.expressed.exon.info %>% filter(`exon_UTR_id` == "exon2")

expressed.gene.only.one.exon <- subset(tgene.expressed.exon.info , !(gene.id %in% expressed.gene.exon_2$gene.id))

# filter positive result with one intron (2 exon)

tgene.expressed.exon3 <- tgene.expressed.exon.info %>% filter(`exon_UTR_id` == "exon3")

tgene.expressed.with1and2exon <- subset(tgene.expressed.exon.info, !(gene.id %in% tgene.expressed.exon3$gene.id))

tgene.expressed.with2exon <- subset(tgene.expressed.with1and2exon, !(gene.id %in% expressed.gene.only.one.exon$gene.id)) %>% filter(`exon_UTR_id` == "exon2")

For dicistronic PCGs candidates with one or no introns, use the intron coordinates from the nearest gene as background read coverage control.

# Find the nearest genes:

bedtools closest -a No_intron_genes.txt \

-b ALL_introns_info.txt \

-k 2 > No_Intron_closest2_Introns.bed

bedtools closest -a One_intron_genes.txt \

-b ALL_introns_info.txt \

-k 2 > One_Intron_closest2_Introns.bed

After finding the nearest introns of these tRNA-mRNA loci candidates, go back to the statistical test for the average depth.

**4. Evaluate the continuous read coverage of the intergenic region.** To identify dicistronic transcripts, examine the RNA read coverage at single-base resolution across the intergenic region between the tRNA gene and the coding sequence (CDS) of the adjacent protein-coding gene (PCG).

Classify candidates according to the continuity of read coverage across this intergenic region. The DiRT v2 pipeline assigns two labels to candidates that exhibit continuous coverage:

High-confidence candidates (continu.cov = TRUE).

Assign this label to candidates that display complete intergenic coverage across every base pair in all biological replicates independently.

Merged-sample candidates (combined.continu.cov = TRUE).

Because Illumina RNA-seq coverage can vary substantially with sequencing depth, pool reads from all replicates and re-evaluate coverage across the intergenic region. Assign this label to candidates that show continuous coverage across every base pair in the merged dataset, even if full coverage is not observed in each replicate individually.

allIntergenic <- names(coverage.intergenic$RNA.sorted.bam) # pull out sample name from any sample

allSamples <- names(coverage.intergenic)

coverage.samples <- lapply(allIntergenic,function(intergenic){

 v <- lapply(allSamples, function(sample){

 coverage.intergenic[[sample]][[intergenic]] %>% as.integer()

 })

 v <- do.call(rbind,v)

 rownames(v) <- allSamples

 return(t(v))

})

names(coverage.samples) <- allIntergenic

coverage.list.final <- nZero.intergenic %>%

 merge(final.stats.parid, by = "ids") %>%

 filter(id %in% final.sign) %>%

 mutate(continu.cov = rowSums(dplyr::select(., contains("nZero"))) == 0)

# make all coverage data addable for each sample and assess the continious Cov for combined coverage

keptIntergenic <- coverage.list.final$ids

coverage.samples.final <- coverage.samples[names(coverage.samples) %in% keptIntergenic]

combined.nZero <- coverage.samples.final %>%

 lapply(function(intergenic){

 sum(rowSums(intergenic)==0)

 }) %>% unlist() %>% as.data.frame() %>% rownames_to_column() %>%

 set_colnames(c("ids","Combined.nZero"))

continuous.coverage.final <- left_join(coverage.list.final,combined.nZero, by = "ids") %>%

 mutate(Combined.nZero = Combined.nZero == 0) %>%

 setnames("Combined.nZero", "combined.continu.cov")

continuous.coverage.final.all <- rbind(

 continuous.coverage.final %>% mutate(num.Intron = "2 and more"),

 continuous.coverage.final_One_intron %>% mutate(num.Intron = "1"),

 continuous.coverage.final_No_intron %>% mutate(num.Intron = "no")

) %>%

 filter(combined.continu.cov == TRUE)

**5. Statistical testing for intergenic and introns read coverage.** To test if the RNA-seq read coverage of the intergenic region is higher than background noise, use introns as the comparative control (Student’s t-test). Student’s t-test was used in DiRT v1 [3] and in v2 as the recommended method for read-coverage comparison between genomic loci, as well as in intron retention analyses to distinguish true continuous transcripts from pre-mRNA processing intermediates or to detect genomic DNA contamination noise [25,26]. To account for sample variation and background sequencing noise, RNA-seq data representing a minimum of three biological replicates is recommended for this statistical test.

# statistical test

# Map the tRNAs to genes

tRnaToGene <- aveCoverage.final %>%

 filter(grepl("t[rR][nN][aA]", ids),

 !is.na(aveCoverage)) %>%

 distinct(ids, .keep_all = TRUE) %>%

 dplyr::select(contains("id")) %>%

 mutate(id = as.character(id)) %>%

 split(f = .$id) %>%

 lapply(function(x){

 if (nrow(x) == 1) return(x) # This removes the genes with >1 tRNA, already fixed with unique tRNA-gene combo id

 }) %>%

 bind_rows()

# create a list with 2 elements with different comparison and do the t.test

tTestResults.final <- tRnaToGene$ids %>%

 # extract(1) %>%

 lapply(function(x){

 # Get the geneid & coverage as a matrix

 combo <- filter(tRnaToGene, ids ==x)$id

 cov <- filter(aveCoverage.final, id == combo) %>%

 acast(Sample~type, value.var = "aveCoverage")

 #Initialise the output

 out <- data_frame(

 ids = character(),

 id = character(),

 comparison = character(),

 df = double(),

 V = double(),

 p = double()

 )

 # Compare the introns

 if (all(c("intron_2nd_closest_to_intergenic", "intron_closest_to_intergenic") %in% colnames(cov))) {

 tTest <- t.test(log(cov[,"intron_closest_to_intergenic"]+1),

 log(cov[,"intron_2nd_closest_to_intergenic"]+1),

 paired = TRUE)

 out %<>%

 bind_rows(

 data_frame(ids = x,

 id = combo,

 comparison = "intron_2nd_closest_to_intergenicVsintron_closest_to_intergenic",

 df = tTest$parameter, # degree of freedom

 V = tTest$statistic,

 p = tTest$p.value)

 )

 }

 # Compare the intergenic regions

 if (all(c("intergenic", "intron_closest_to_intergenic") %in% colnames(cov))) {

 tTest <- t.test(log(cov[,"intron_closest_to_intergenic"]+1),

 log(cov[,"intergenic"]+1),

 paired = TRUE)

 out %<>%

 bind_rows(

 data_frame(ids = x,

 id = combo,

 comparison = "IntergenicVsintron_closest_to_intergenic",

 df = tTest$parameter,

 V = tTest$statistic,

 p = tTest$p.value)

 )

 }

 out

 }) %>%

 bind_rows() %>%

 mutate(adjP = p.adjust(p, "bonferroni"),

 FDR = p.adjust(p, "fdr")) %>%

 arrange(p) %>%

 split(f = .$comparison)

# t.test output

# for FDR

intergeic.sign.final <- tTestResults.final$IntergenicVsintron_closest_to_intergenic %>%

 filter(FDR < 0.05, V < 0)

intron.not.sign.final <- tTestResults.final$intron_2nd_closest_to_intergenicVsintron_closest_to_intergenic %>%

 filter((FDR < 0.05 & V > 0) | FDR >=0.05)

final.sign <- intersect(intron.not.sign.final$id,intergeic.sign.final$id)

**6. Assign the intron count for each dicistronic transcript.** In the result data sheet, add a column to label how many introns are in the dicistronic PCGs.

{R}

continuous.coverage.final.all <- rbind(

 continuous.coverage.final %>% mutate(num.Intron = "2 and more"),

 continuous.coverage.final_One_intron %>% mutate(num.Intron = "1"),

 continuous.coverage.final_No_intron %>% mutate(num.Intron = "no")

) %>%

 filter(combined.continu.cov == TRUE)

**7. Determine whether the tRNA resides within the mRNA UTR by comparing its genomic coordinates to the full gene coordinates, instead of using CDS coordinates.**

# 1. Add Gene information to DT result

#Gene information

gene.information <- gene.info.from.gff %>%

filter(gene.biotype == "gene") %>%

mutate(gene.id = sub(".*ID=([^;]+).*", "\\1", gene.id)) %>%

arrange(gene.chr, gene.end)

DT_with_Gene_Startend <- Dicistronic.all.tRNA %>%

left_join(gene.information, by = "gene.id")

DT_Final_Classified <- DT_with_Gene_Startend %>%

mutate(

tRNA_in_Gene = (tRNA.start >= gene.start & tRNA.end <= gene.end),

tRNA_in_CDS = (tRNA.start >= CDS.start & tRNA.end <= CDS.end),

Location_Type = case_when(

tRNA_in_CDS == TRUE ~ "CDS-Internal",

tRNA_in_Gene == TRUE & tRNA_in_CDS == FALSE ~ "UTR_or_Intron",

(tRNA.start < gene.end & tRNA.end > gene.start) & tRNA_in_Gene == FALSE ~ "Gene-Boundary-Overlap",

TRUE ~ "Intergenic"))

# The final output file: Expected to have columns as:

Id (the dicistronic id combine with tRNA+gene), chr, start (whole transcript), end(whole transcript), gene.id, CDS.start, CDS.end, gene.strand, tRNA.id, tRNA.start, tRNA.end, tRNA.strand, tRNA.upstream (from gene strand), tRNAFirst (from genome coordinate), interval.length, num.Intron, combined.continu.cov, intergenic gap in each sample, gene.start(including UTR), gene.end (including UTR), Location_Type (tRNA in UTR or not)

write.table(DT_Final_Classified,

 file = "Final_Result_withIntronNmb.xlsx",

 quote = FALSE, sep = "\t", row.names = FALSE

Finally, to assist in validation of the transcriptomic continuity of the statistically significant dicistronic candidates, load the pipeline-generated dicistronic transcripts PCG ID and alignment bam files into the Integrative Genomics Viewer (IGV) [27] to manually review the per-base read coverage across the tRNA, intergenic, and mRNA loci.

The full code of the DiRT v2 pipeline is accessible in the GitHub link:

https://github.com/AdelaideUniPlantMobileRNA/DiRT_Pipeline_V2

**C. Examples of implementation of DiRT v2**

Using DiRT pipeline v2, we identified dicistronic transcripts from two different Illumina-based RNA-seq datasets (Table 3). Data from example 1 below is an in-house-generated RNA-seq dataset from leaves collected from the hybrid grapevine cultivar Ruggeri-140 (*Vitis berlandieri* × *V. rupestris*). Data from example 2 below uses public Illumina RNA-seq data from *Arabidopsis* 14-day-old whole seedling samples (PRJEB32714).

**C1. Identification of dicistronic transcripts in grapevine (example 1)**

***Sampling material, RNA isolation, RNA-sequencing, and tRNA-mRNA transcript in silico prediction***

To identify dicistronic transcripts from grapevine RNA sequencing data, potted grapevine Ruggeri-140 was grown in glasshouse conditions for two months. Young leaf tissue from four biological replicates was collected and immediately snap-frozen in liquid nitrogen. Total RNA was extracted using the Spectrum Plant Total RNA kit following the standard protocol. NEBNext^®^ Poly(A) mRNA magnetic isolation module (New England Biolabs) was used for poly-A enrichment, and RNA-seq library was prepared using the NEBNext^®^ Ultra^TM^ II Directional RNA library prep with sample purification beads (New England Biolabs). Resulting libraries were individually indexed, equimolarly pooled, and sequenced on the platform Illumina NovaSeq X Plus (PE150-25B), yielding an average of 60 million 150 bp paired-end reads for each sample. Data was processed as explained in the Procedure section above. The CleanUpRNAseq tool [24] indicated very low levels of genomic DNA contamination across all samples (mean 1.5%, Supplemental Table 4). For the poly-A-enriched stranded RNA-seq data used here, such low levels are attributed to background sampling noise and therefore do not require correction [24]. The *Vitis vinifera* T2T reference genome v5 [19] was used for mapping, genomic feature annotation, and identification of Trna-mRNA transcripts.

**C2. Identification and validation of dicistronic transcripts in *Arabidopsis thaliana* (example 2)**

***Data acquisition and tRNA-mRNA transcript in silico prediction***

*Arabidopsis thaliana* RNA-seq data were downloaded from the Sequence Read Archive (SRA) of the National Center for Biotechnology Information (NCBI) under BioProject PRJEB32714 (14-day-old whole seedling samples grown under constant 21 °C long-day cycles). Total RNA was extracted with the RNeasy Plant Mini kit (Qiagen) and was poly-A-enriched. Directional RNA sequencing was conducted with the Illumina 2000 system. Data was processed as described above using the *A. thaliana* genome TAIR10.1 [21] for sequencing read mapping, genomic feature annotation, and identification of tRNA-mRNA transcripts.

**C3. Validation of dicistronic tRNA-mRNA candidate using semi-quantitative RT-PCR**

To experimentally validate the dicistronic transcript candidate identified from the public *Arabidopsis* RNA-seq dataset PRJEB32714, we performed semi-quantitative RT-PCR for the candidate tRNA-GlyGCC_AT1G71697. Total RNA was extracted from *Arabidopsis thaliana* Col-0 wild-type samples using the Spectrum Plant Total RNA kit following the standard protocol. First-strand cDNA was synthesized using the SuperScript^TM^ III first-strand synthesis system following the manufacturer’s manual. To control for potential genomic DNA contamination, a negative control lacking reverse transcriptase (-RT) was prepared in parallel with the cDNA synthesis reactions. PCR amplification was performed using DreamTaq PCR master mix for 35 cycles with an annealing temperature of 57 °C. Primers were designed within the tRNA-Gly sequence and the second exon of the adjacent protein-coding gene AT1G71697, spanning a 101 bp intron to distinguish amplification from cDNA vs. genomic DNA templates. Primer sequences are provided in Supplemental Table 3. The *Arabidopsis* gene *PDF2* (AT5G44430) was used as the internal expression of the housekeeping gene for the RT-PCR assay.

## Data analysis

**Result interpretation**

**DiRT v2 implementation in grapevine (*Vitis* Ruggeri-140) and *Arabidopsis thaliana***

**Figure 2.**
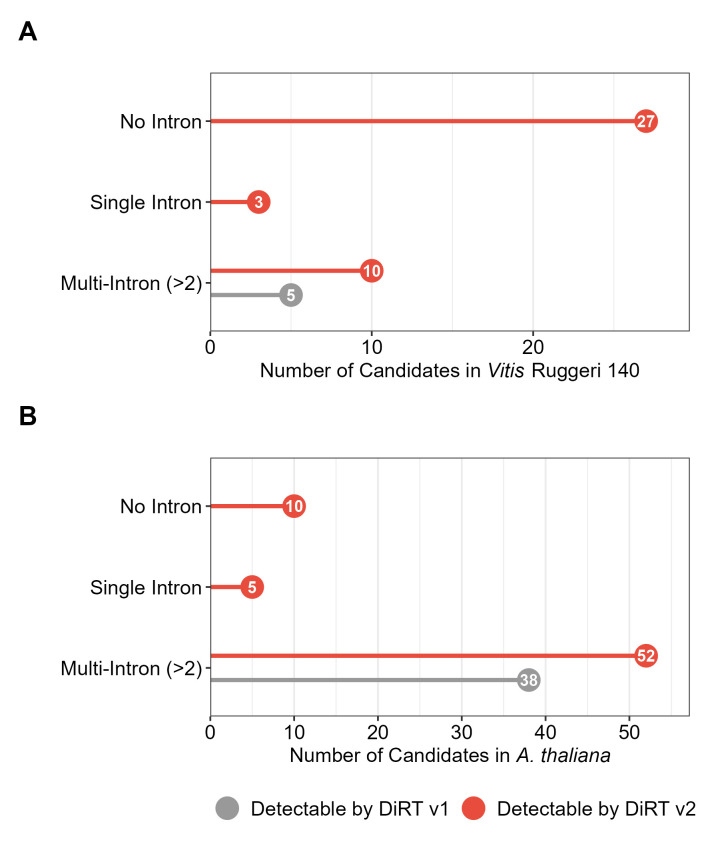
Detection of dicistronic tRNA-mRNA transcripts in grapevine and *Arabidopsis* RNA-seq datasets using DiRT v2 compared to DiRT v1 pipeline [3]. (A) Dicistronic tRNA-mRNA candidates identified by DiRT v2 (red) vs. the legacy DiRT v1 (gray) pipeline for *Vitis* Ruggeri 140 leaf samples [3]. (B) Dicistronic tRNA-mRNA candidates identified by DiRT v2 (red) vs. the legacy DiRT v1 (gray) pipeline for *Arabidopsis thaliana* whole seedling samples. In both species, the updated DiRT v2 detected additional dicistronic candidates, including intronless, single intron, and multi-intron transcripts, compared to DiRT v1.

**Figure 3.**
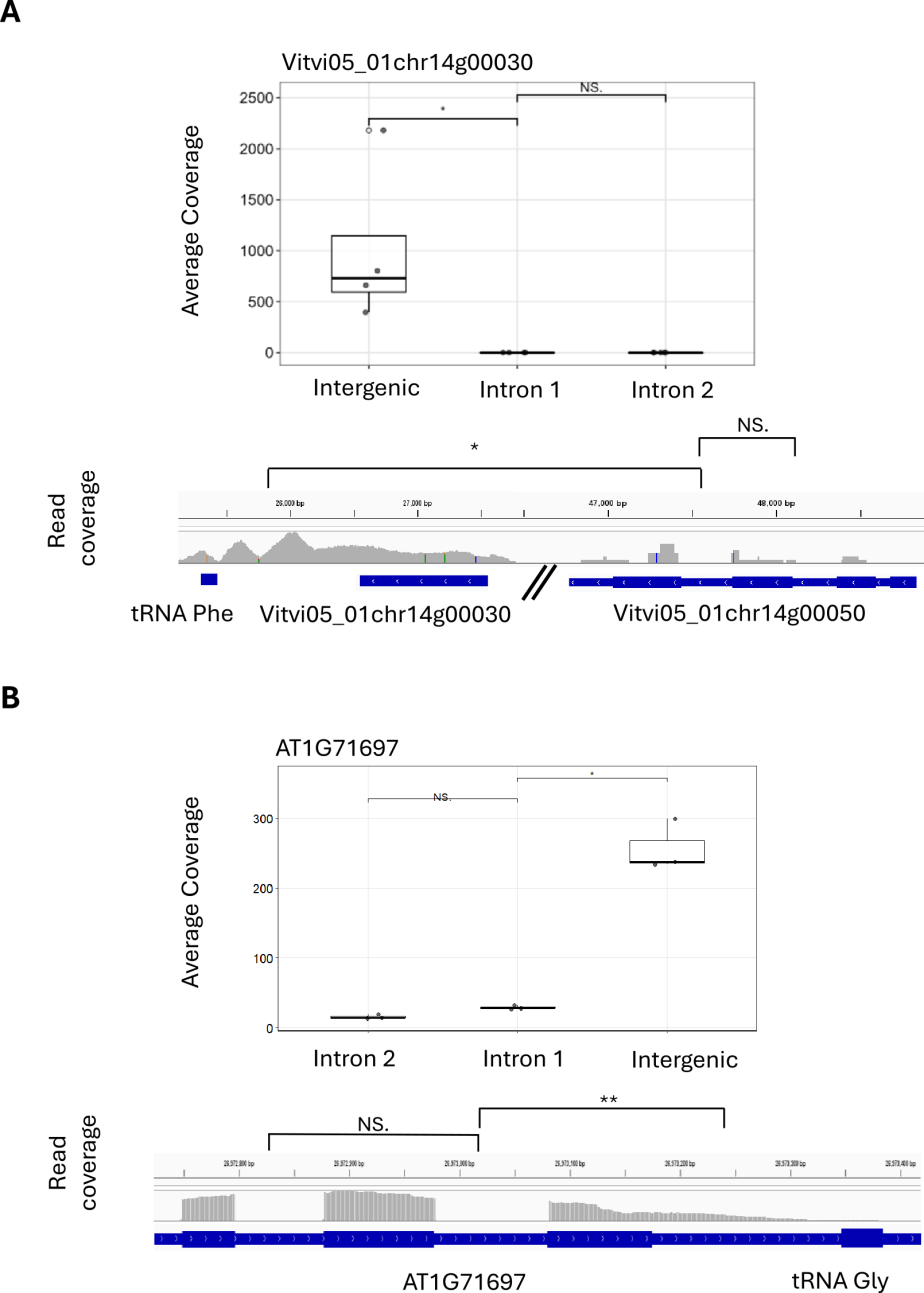
Integrative Genomics Viewer (IGV) image of dicistronic tRNA-mRNA transcripts identified with the DiRT v2 pipeline, with box plots showing the statistical test of each candidate. (A) Representative example of a dicistronic transcript detected in grapevine RNA-seq samples (tRNA-Phe_Vitvi05_01chr14g00030). The top box plot displays the average RNA read coverage, while the bottom panel shows the corresponding IGV track. Because the adjacent protein-coding gene (PCG) lacks introns, intronic regions from the nearest neighboring gene (Vitvi05_01chr14g00050) were used as a background control. Intergenic read coverage is significantly higher than control Intron 1 (FDR = 0.002), with no significant difference between control Introns 1 and 2 (FDR = 0.73). (B) Representative example of a dicistronic transcript candidate (tRNA-Gly_AT1G71697) identified in the *Arabidopsis thaliana* public RNA-seq dataset (PRJEB32714). The tRNA sequence is located in the 3' UTR of the adjacent PCG. Read coverage in the intergenic region is significantly higher than in Intron 1 (FDR = 0.0086), whereas Intron 1 coverage is not significantly different from Intron 2 (FDR = 0.075).

Evolutionary studies of plant genomes reveal that a significant proportion of plant genes have lost their introns [5]. In the grapevine genome, 1,054 protein-coding genes have a tRNA gene up or downstream, and 407 of these genes have a single intron or are intronless. These potential dicistronic tRNA-mRNA candidates were not analyzed using the first version of the DiRT v1 pipeline. Using the DiRT v2 pipeline, we identified 40 dicistronic tRNA-mRNA transcripts from a grapevine *Vitis* Ruggeri leaf sample (Figure 2A, Table 4, and Supplemental Table 1). The new version of the pipeline revealed that 27 candidates do not have introns (representative candidate shown in Figure 3A), 3 of which contain a single intron, and 10 candidates contain more than 2 introns. Furthermore, 7 candidates contain the tRNA/TLS located within the UTR of the PCG. Notably, of the 40 candidates, only 5 of them can be identified with the previous version of the DiRT v1 pipeline (Figure 2A and Table 4).

**Table 4.** Comparison of DiRT v1 [3] vs. DiRT v2 output for *Vitis* Ruggeri 140 and *A. thaliana*

Species	DiRT version	Intron number			tRNA/TLS localization	
		Intronless	Single intron	Multi-intron	Intergenic	UTR
*Vitis* Ruggeri 140	DiRT v1	0	0	5	5	0
	DiRT v2	27	3	10	33	7
*A. thaliana*	DiRT v1	0	0	38	38	0
	DiRT v2	10	5	52	51	16

To further evaluate the applicability of the DiRT v2 pipeline, we tested it using publicly available *Arabidopsis thaliana* Illumina RNA-seq data (PRJEB32714). Using this dataset, we identified 67 dicistronic transcripts (Figure 2B, Table 4, and Supplemental Table 2): 52 contained two or more introns, 5 contained a single intron, and 10 were intronless. Notably, 38 of the 67 dicistronic transcripts were detected using the legacy DiRT v1 criteria, while the remaining 29 candidates are uniquely captured by DiRT v2 (representative candidate shown in Figure 3B). This highlights the key improvement in the DiRT v2 pipeline to reliably detect dicistronic tRNA-mRNA transcripts in *A. thaliana* and grapevine, confirming the pipeline’s validity and robustness across multiple plant species to characterize these novel RNA features.

## Validation of protocol

**Figure 4.**
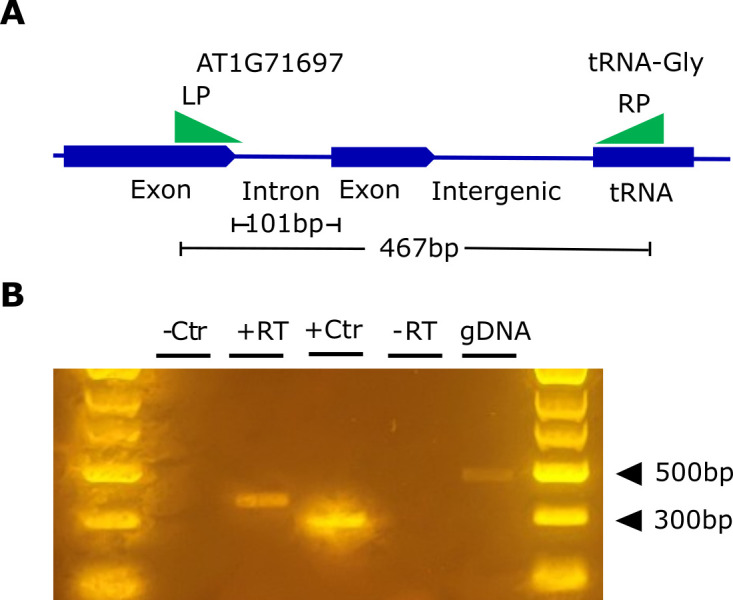
Semi-quantitative RT-PCR confirmation of dicistronic transcripts tRNA-Gly_AT1G71697. (A) Schematic figure of the target dicistronic transcripts in *Arabidopsis thaliana* gene AT1G71697 showing primers used (green triangles) for RT-PCR. (B) RT-PCR confirmation of actively transcribed intergenic region between tRNA and At1g71697 mRNA. +RT cDNA used as template, genomic DNA (gDNA) used as a control, -RT (minus reverse transcriptase) PCR negative control, and +Ctr *PDF2* (AT5G44430) used as an internal expression of a housekeeping gene. Full length of RT-PCR amplicon is 366 bp (+RT), while the expected gDNA PCR amplicon is 467 bp (gDNA), and the *PDF2* amplicon is 297 bp (+Ctr).

The validation of dicistronic transcripts in grapevine has been conducted with candidates identified from DiRT v1 [3]. We experimentally validated the candidate tRNA-GlyGCC_AT1G71697 identified from *Arabidopsis thaliana* public RNA sequencing data PRJEB32714 using semi-quantitative RT-PCR. Primers were designed in the PCG second exon and the tRNA sequence, spanning a 101 bp intron. The reverse-transcribed RNA (+RT) produced a 366-bp product, matching the predicted size of the amplicon. The genomic DNA (gDNA) control produced the expected 467-bp product, which included the intervening intron (Figure 4).

## General notes and troubleshooting

**Troubleshooting**

**Problem 1:** The CDS coordinates generate an empty dataframe (section B1).

Possible cause: The gene ID in different GFF annotations may vary.

Solution: Gene annotation used for the reference genome alignment should be a standard gff3 file.

**Problem 2:** When using the bedtools closest function, the result file may be empty (section B1).

Possible cause: The .txt files have Windows-style CR-LF line endings.

Solution: Use notepad++ (*Edit* > *EOL conversion* > *Unix*) to fix them. If bedtools require sorted input files, use this code to sort the files:

sort -k1,1 -k2,2n CDS.start.end.for.gene.txt > CDS.start.end.for.gene.sorted.txt

sort -k1,1 -k2,2n tRNA.info.txt > tRNA_sorted.txt

sort -k1,1 Reference_genome.txt > Reference_genome_sorted.txt

**Problem 3:** Computing environment issue.

Possible cause: Although the workflow is designed to be portable across computing environments through Nextflow, users may encounter installation challenges related to operating system–specific dependencies, software versions, or local system configurations.

Solution: The latest installation instructions, updates, and troubleshooting guidance are maintained in the project’s GitHub repository: https://github.com/AdelaideUniPlantMobileRNA/DiRT_Pipeline_V2.

## Supplementary information

The following supporting information can be downloaded here:

1. Supplemental Table 1. Dicistronic tRNA-mRNA transcripts identified from *Vitis* Ruggeri 140 leaf samples.

2. Supplemental Table 2. Dicistronic tRNA-mRNA transcripts identified from the *Arabidopsis thaliana* dataset PRJEB32714.

3. Supplemental Table 3. Primer pair for RT-PCR.

4. Supplemental Table 4. Genomic DNA contamination calculation in grapevine samples.
